# Reduction of breakdown threshold by metal nanoparticle seeding in a DC microdischarge

**DOI:** 10.1186/s11671-014-0709-y

**Published:** 2015-01-28

**Authors:** Jordan Sawyer, Jacques Abboud, Zhili Zhang, Steven F Adams

**Affiliations:** Department of Mechanical, Aerospace and Biomedical Engineering, University of Tennessee, Knoxville, TN 37996 USA; Air Force Research Laboratory (AFRL/RQQE), Wright-Patterson AFB, Dayton, OH 45433-7919 USA

**Keywords:** Microdischarge, Metal nanoparticles, Threshold

## Abstract

Significant reduction of the breakdown threshold in a DC microdischarge via seeding metal nanoparticles has been demonstrated. Compared to standard Paschen curves in dry air, reductions in the breakdown voltage of 5% to 25% were obtained for *PD* values (the product of pressure and electrode gap distance) ranging from 20 to 40 Torr-cm by seeding aluminum and iron nanoparticles with mean sizes of 75 nm and 80 nm, respectively. No secondary energy source was required to achieve this breakdown threshold reduction. From high-speed chemiluminescence imaging of the discharge evolution, breakdown was shown to be initiated at reduced voltages. Following breakdown, the increase in temperature ignited some of the nanoparticles near the cathode. Results suggest that possible charging of the nanoparticles within the gap may reduce the effective transient distance, leading to the threshold reduction.

## Background

The mechanisms for breakdown in gas discharges have been studied extensively for over a century [1]. It is well known that the voltage required to initiate breakdown of a gaseous DC discharge depends strongly on the pressure *P* multiplied by the distance *D* of the gap between the electrodes, *PD*, as described by Paschen's Law [2]. For a gas at atmospheric pressure, by limiting the inter-electrode separation to a distance of less than a millimeter, it is possible to produce a stable ‘normal-glow’ microdischarge [3]. Although such a microdischarge is spatially confined compared to a traditional low-pressure discharge, the normal glow properties of a nonthermal discharge still apply, such as an electron temperature, *T*e, which is several orders of magnitude larger than the gas temperature, *T*g [3-5]. Many applications have been explored to exploit this excess electron energy and drive optical or chemical processes such as vacuum-ultraviolet light sources [6], biomedical systems [7,8], nanoparticle synthesis [9,10], and plasma ignition [11]. However in atmospheric air, even with a small inter-electrode separation of less than 1 mm, a sizeable voltage (upwards of 4 to 5 kV) may still be required to initiate breakdown.

A significant reduction of the breakdown threshold within a microdischarge could be a breakthrough that enables numerous applications. Such a breakdown voltage reduction could allow for the use of smaller, cheaper, and safer power supplies. One method of reducing the voltage required to initiate breakdown is to generate seed electrons into the discharge region. Breakdown under these conditions is termed ‘under-voltage breakdown’. Previously studied techniques for achieving under-voltage breakdown have included electron seeding by illumination of the cathode by ultraviolet (UV) light [12-14], resonance enhanced multi-photon ionization (REMPI) by UV pulsed lasers [15,16], and the use of secondary electrodes or spark plugs [17]. The major limitation of these methods is that they all require a secondary energy source in order to produce the seed electrons which can increase the overall cost, complexity, and weight of the microdischarge system.

On the other hand, the effect of solid particle contamination, whether intentionally introduced or not, on the breakdown process in air gaps has been explored previously. Unwanted solid particle contaminants in commercial electrical systems can lead to arcing and failure of transmission lines and gas insulation systems. Sand and dust with particle sizes above several tens of microns were shown to initiate breakdown across gap lengths of several centimeters [18,19]. The previous works concluded that the sand and dust particles in the inter-electrode gap played a negligible role in the volume processes during breakdown; however, the formation of a thin contaminant layer on the cathode enhanced secondary electron emission and significantly reduced the breakdown voltage in some circumstances. Other works have shown that larger (100 s of microns to 100 s mms) conductive particles in an inter-electrode air gap can play a significant role in reducing breakdown voltage, time-lag of impulses, and breakdown probability [20-22]. These previous works have concluded that the degree of influence that the solid particles have on the breakdown process depends on both the discharge properties, namely polarity and field uniformity, as well as properties of the particles such as conductivity, shape, size, concentration, and position relative to the electrodes.

In this study, aluminum and iron nanoparticles were seeded into a DC microdischarge at a very low flow speed. Experimental results indicate that breakdown thresholds of dry air with nanoparticle seeding were significantly reduced compared to standard Paschen law. High-speed chemiluminescence images reveal that the nanoparticles had a major influence in inducing breakdown and subsequent heating that eventually ignited the nanoparticles.

## Methods

**Figure 1:**
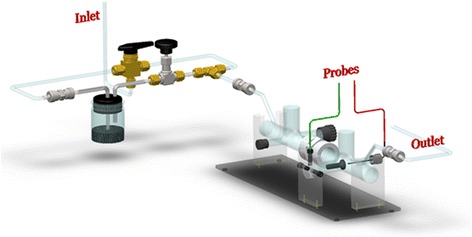
**Discharge cell setup for the flow system and particle seeder.**

The experimental setup consisted of three major components: 1) a microdischarge cell with a gas and particle handling system that produces a DC microdischarge either with or without nanoparticle seeding, 2) a high-voltage power supply, high-voltage probes, and data acquisition systems, and 3) a high-speed camera for luminosity measurements. In Figure 1, a home-made glass cell (inner diameter = 1 in.) was equipped with two adjustable electrodes. The cathode was a circular aluminum plate while the anode was a stainless steel needle oriented orthogonally to the circular plane of the cathode. The cathode was attached to the head of a micrometer so that its position relative to the anode could be accurately controlled. The distance between the two electrodes was set to roughly zero as verified by measuring the resistance across the gap with a multimeter. The gap between the electrodes could be determined accurately down to a 0.03 mm resolution.

A custom-made particle seeder, which included a particle container, inlet and outlet tubes, was used to seed the flow with various nanoenergetic materials. Mass measurements, before and after each run, were used to estimate the particle flow rates. Usually about 5 g of aluminum or iron nanoenergetics (nominal diameters of 70 nm, purchased from NanoAmor Inc., Houston, USA, without further treatment) were placed along the flow path inside the particle container. The particle seeder inlet tube was submerged below the surface of the piles to generate particle suspensions inside the container. The height of the inlet and outlet were offset to ensure roughly uniform seeding in the flow through the discharge tube. Standard breathing dry air was used as the gas supply. A flow controller (FMA 5400, Omega, Stamford, USA) regulated the flow rate in the system to 1 ± 0.1 standard liters per minute (slpm). A three-way valve allowed the flow to be switched between the nanoparticle seeder and a bypass line. If particle seeding was desired, the three-way valve and needle valve were opened to allow flow through the seeder. Otherwise, the three-way valve and needle valve were closed to isolate the particle seeder. The pressure in the system was monitored downstream of the cell by a piezoelectric pressure sensor (Series 910, Kurt J. Lesker Company, Jefferson Hills, USA), and the desired pressure was obtained by adjusting the needle valve upstream of the vacuum pump.

The high-voltage system included a high-voltage power supply, high-voltage probes, and data acquisition systems. The output of the high-voltage power supply was controlled via Labview™ software on a personal computer. The positive terminal was connected to a 1 MΩ ballast resistor used to limit the current through the discharge circuit. When a sufficient positive voltage was applied to the anode, breakdown occurred within the air between the two electrodes, with the resulting current spike traveling through the circuit to the grounded cathode. The supply voltage was linearly increased until breakdown was achieved. Two high-voltage probes were used to monitor the voltage drop across the electrode gap and across the ballast resistor. The voltage drop across the electrode gap, *V*_1_, was determined directly by measuring the potential at junctions before the anode and after the cathode. The current in the system was obtained by measuring the potential drop across the ballast resistor and electrode gap sequentially. The total applied potential across the ballast and gap could be expressed as *V*_2_ = *V*_1_ + *V*_R_. Rearranging and dividing by the resistance of the ballast resistor, *R*, the current in the circuit was determined by Ohm's Law to be *I* = (*V*_2_ − *V*_1_)/*R*.

A high-speed camera (HS1200, Cooke Corporation, Kelheim, Germany) was used to track the chemiluminescence from the plasma initiation and nanoparticle ignition events. The 2D chemiluminescence images were acquired with a 1 ms exposure time. Because of the nature of the volume-averaged chemiluminescence images, the measurements can be regarded as an average over the line of sight across the discharge.

## Results and discussions

**Figure 2:**
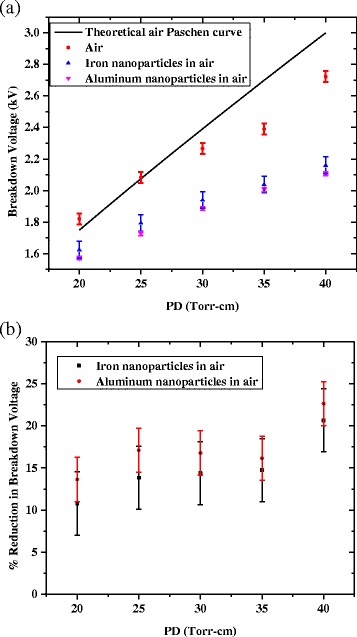
**Comparison between theoretical Paschen curves for ‘clean’ dry air and experimental measurements in air. (a)** Comparison of Paschen curves for ‘clean’ dry air and dry air with nanoparticle seeding with error bars corresponding to one standard deviation. **(b)** Percent reduction in breakdown voltage from ‘clean’ dry air with aluminum and iron nanoparticle seeding with error bars corresponding to one standard deviation.

Figure 2 shows a comparison between theoretical Paschen curves for ‘clean’ dry air and experimental measurements in air with and without Al and Fe nanoparticle seeding. Measurements of the breakdown voltage of the ‘clean’ dry air were taken for a 1-mm gap distance with varying pressures of 200 to 400 Torr. For the dry air case, as seen in Figure 2, a maximum error of less than 12% was observed between theoretical and experimental breakdown voltages. Breakdown voltages for the dry air with nanoparticle seeding were experimentally determined for the 20 to 40 Torr-cm range. The experiment was performed with aluminum and iron nanoparticles with mean diameters of 80 and 75 nm, respectively. The number density flow rate of the nanoparticles had an upper bound of 1 × 10^10^ cm^−3^ s^−1^ during the course of the experiment. The breakdown threshold values for all three curves appear to coalesce at the lower *PD* scaling close to 20 Torr-cm; however, the experimental data diverge from the theoretical values with increasing *PD* up to 40 Torr-cm. This situation is most commonly due to uncertainty in the secondary ionization coefficient of the cathode. It is clearly seen that nanoparticle seeding resulted in a reduction in the breakdown voltage for the entire 20 to 40 Torr-cm range. However, at lower *PD* values, nanoparticle seeding appeared to cause only a slightly reduced breakdown threshold of a few percent compared to the ‘clean’ dry air case. On the contrary, at larger *PD* values, a more significant reduction in the breakdown voltage, approaching 25%, was apparent with nanoparticle seeding. In Figure 2 a general upward trend is seen in the percentage reduction for increasing *PD* for the 20 to 40 Torr-cm range. Seeding with Al nanoparticles consistently has a lower breakdown threshold compared to seeding with Fe nanoparticles. This may be due to the difference in electrical conductivity of the particles. Additionally, iron nanoparticles have been shown to have larger more porous oxidation layers which may negatively impact charging of the nanoparticles [23]. Mild day to day variations were seen in the experimental breakdown voltages most likely due to changes in the electrode surfaces over time despite rigorous efforts in polishing and cleaning; however, the abovementioned trends were consistently preserved.

**Figure 3:**
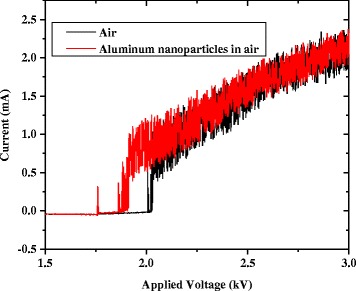
**Discharge current versus applied voltage with and without seeding of aluminum nanoparticles at PD = 25 Torr-cm.**

**Figure 4:**
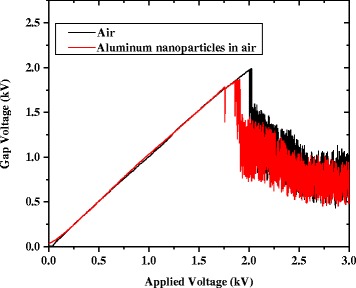
**Gap voltage versus applied voltage with and without seeding of aluminum nanoparticles at PD = 25 Torr-cm.**

Figure 3 shows a typical plot of the discharge current as the applied voltage was gradually increased, both with and without Al nanoparticle seeding. Initially, in both cases, there is negligible current in the circuit prior to breakdown. Without nanoparticle seeding, there is an almost immediate sharp increase in current at the breakdown voltage. With nanoparticle seeding, short-lived instabilities manifest as peaks in current just prior to breakdown. A slightly elevated current is present as well in the instability region. Once the breakdown threshold was reached in each discharge, there was negligible difference in current at similar applied voltages. Similar properties are shown for the measured gap voltage, with and without aluminum nanoparticle seeding, as shown in Figure 4. Without nanoparticle seeding, there is a sharp decrease in voltage across the gap at the breakdown voltage. For the case with nanoparticle seeding, instabilities appear as dips in the voltage just prior to breakdown. Once the breakdown threshold is reached, it is apparent from the two plots that the nanoparticle seeding causes negligible change to conductivity of the sustained discharge.

**Figure 5:**
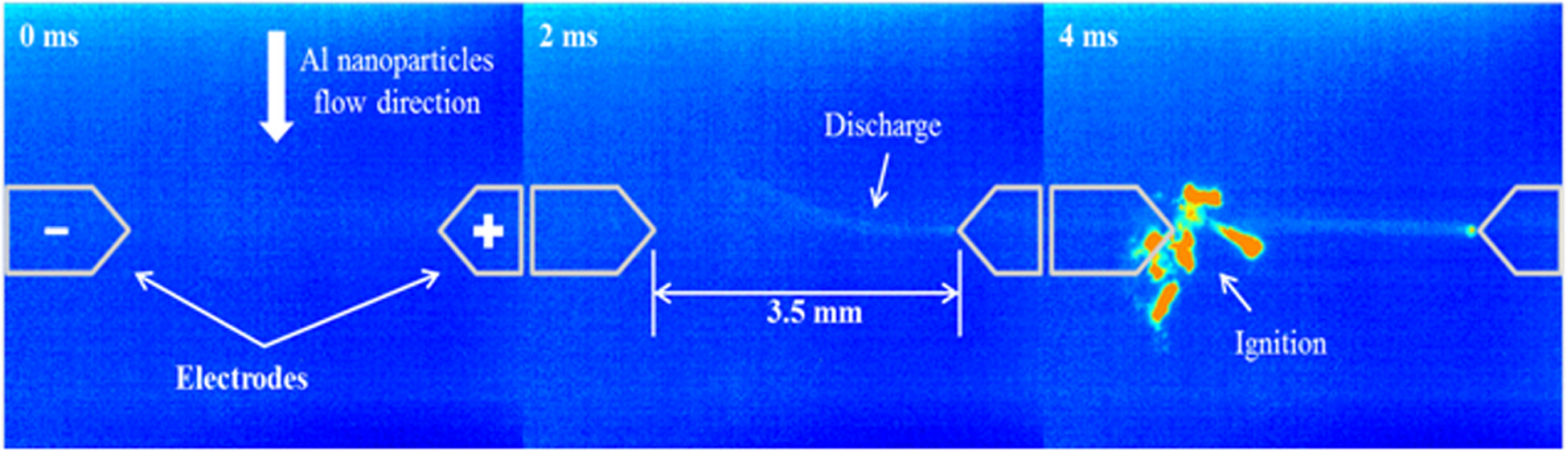
**High-speed chemiluminescence images of breakdown in a 3.5-mm gap in atmospheric air.**

Figure 5 shows high speed images of the discharge gap taken during the breakdown process with a resolution of 2 ms between frames. Analysis of these images revealed that the breakdown occurs within the first 2 ms of applying the voltage. Ignition of the nanoparticles due to thermal effects was observed at 4 ms. It was confirmed that breakdown always occurs before the ignition of metal nanoparticles at various conditions. The delay between gas breakdown and the ignition of the nanoparticles varied. In conditions at pressures close to atmosphere and/or larger electrode separations, the thermal ignition of the nanoparticles was both more abundant and less delayed from the breakdown, which was likely due to the higher temperatures under those conditions. Overall, the nanoparticles first acted to reduce the voltage threshold required for gas breakdown. Subsequently the discharge ignited some of the particles between electrodes.

**Figure 6:**
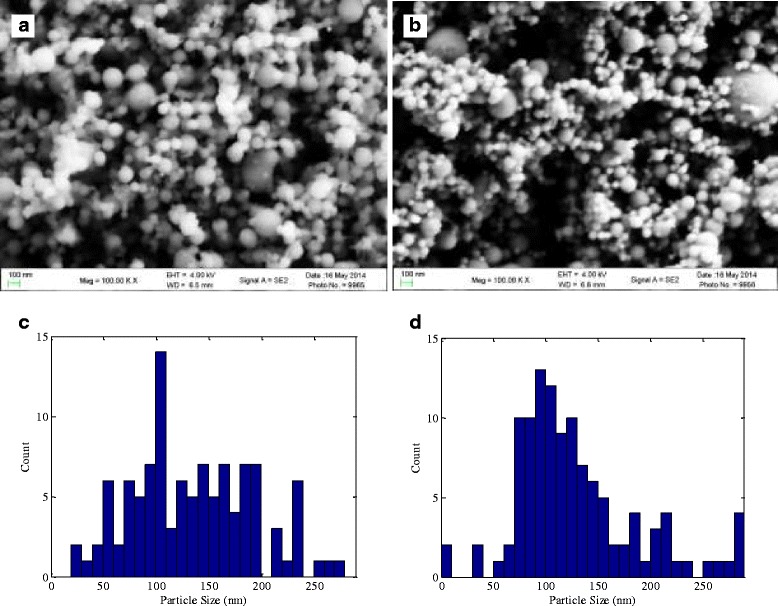
**Scanning electron microscope images and statistical analyses of particle sizes from samples.** SEM images of **(a)** Al and **(b)** Fe nanoparticle samples, and **(c, d)** a statistical analysis of the size distribution of the two samples.

Figure 6 shows scanning electron microscope (SEM) images and statistical analyses of particle sizes from samples of the nanoparticles used in this study. Both the aluminum and iron nanoparticle samples appeared to have Gaussian distributions; however, the mean size of the aluminum nanoparticles is a bit smaller due to the thinner oxidized shell and lower degree of agglomeration. Such *ex situ* diagnostic techniques could be used to build empirical correlations between reduction in breakdown voltage and characteristics of the particles such as size, shape, and degree of oxidation.

Based on the experimental observations, the mechanisms for reducing the breakdown threshold may be due to possible charging of the nanoparticles within the gap, which may reduce the effective distance. Since the current across the gap does not vary with and without nanoparticle seeding, the increased conductivity due to the presence of metal nanoparticles is small. Additionally nanoparticles do not start to combust before the breakdown. The Joule heating due to the presence of metal nanoparticles is slow compared to breakdown generation. However, evidence of charging of the metal nanoparticles within the gap has been observed in these experiments. For example in Figure 5, the attraction of the nanoparticles to the cathode and the subsequent ignition near the cathode region indicate that the nanoparticles were positively charged. In addition, the chemiluminescence image at 2 ms shows a brighter glow near the cathode, which suggests that the evolution of the breakdown started from anode and turned toward the cathode. The nanoparticles charging and shifting toward the cathode might allow the nanoparticles to serve as effective electrodes within the gap, which could mimic a reduction in the gap distance. It is speculated that the Paschen curve may still hold for the particle laden flow with the addition of the conducting nanoparticles near the cathode leading to an effective reduction in distance between the electrodes, i.e., the *PD* values on the x axis are reduced.

## Conclusions

In this work, the seeding of metal nanoparticles was shown to reduce the voltage required to initiate breakdown in an air DC microdischarge. Reductions in the breakdown voltage were seen to be as high as 25% for a *PD* scaling of 40 Torr-cm. High-speed chemiluminescence imaging of the discharge region revealed that the breakdown process was enhanced by a reduction in the required voltage from nanoparticle seeding, and then heating from the discharge led to ignition of some of the nanoparticles as they flowed through the discharge region. The use of SEM imaging gave detailed information regarding the particle size, shape, and oxidation distributions. Further use of *ex situ* diagnostic techniques, such as SEM analyses, could allow for the development of empirical correlations between particle characteristics and reduction in the breakdown voltage. Visual evidence of particle charging being the most likely mechanism for breakdown voltage reduction and subsequent reduction of the effective distance between the electrodes has been presented.
